# Chronic fatigue syndrome patients have alterations in their oral microbiome composition and function

**DOI:** 10.1371/journal.pone.0203503

**Published:** 2018-09-11

**Authors:** Taiwu Wang, Lei Yu, Cong Xu, Keli Pan, Minglu Mo, Mingxiang Duan, Yao Zhang, Hongyan Xiong

**Affiliations:** 1 Department of Epidemiology, College of Preventive Medicine, Third Military Medical University, Chongqing, People’s Republic of China; 2 Research Institute for Medicine of Nanjing Command, Nanjing, P. R. China, Nanjing, China; Medical University of South Carolina, UNITED STATES

## Abstract

Host–microbe interactions have been implicated in the pathogenesis of chronic fatigue syndrome (CFS), but whether the oral microbiome is altered in CFS patients is unknown. We explored alterations of the oral microbiome in Chinese Han CFS patients using 16S rRNA gene sequencing and alterations in the functional potential of the oral microbiome using PICRUSt. We found that Shannon and Simpson diversity indices were not different in CFS patients compared to healthy controls, but the overall oral microbiome composition was different (MANOVA, p < 0.01). CFS patients had a higher relative abundance of Fusobacteria compared with healthy controls. Further, the genera *Leptotrichia*, *Prevotella*, and *Fusobacterium* were enriched and *Haemophilus*, *Veillonella*, and *Porphyromonas* were depleted in CFS patients compared to healthy controls. Functional analysis from inferred metagenomes showed that bacterial genera altered in CFS patients were primarily associated with amino acid and energy metabolism. Our findings demonstrate that the oral microbiome in CFS patients is different from healthy controls, and these differences lead to shifts in functional pathways with implications for CFS pathogenesis. These findings increase our understanding of the relationship between the oral microbiota and CFS, which will advance our understanding of CFS pathogenesis and may contribute to future improvements in treatment and diagnosis.

## Introduction

Bacteria colonize the oral cavity soon after birth and become stable in several niches within this ecosystem. These oral bacterial communities, or microbiome, contain around 1000 different species and are highly complex[1]. The oral microbiome is the second most complex bacterial community in the body after the colon[2]. The oral microbiome is associated with both oral diseases, such as such as tooth decay[3], endodontic infections[4], gingivitis[5], and periodontitis[6], and also with non-oral diseases[7–9]. In a population-based nested case-control study[10], *Porphyromonas gingivalis* and *Aggregatibacter actionmycetemcomitans*, members of the oral microbiome, were associated with an increased risk for pancreatic cancer, while the phylum *Fusobacteria* and its genus *Leptotrichia* were associated with decreased risk for pancreatic cancer. Further, changes in the composition of bacteria present in the oral microbiome are associated with intestinal dysbiosis in murine models of colitis[11]. Additional evidence has demonstrated that the oral microbiome plays an important role not only in disease states but also in human health, including in immune response, metabolism, and nutrient digestion[12, 13].

Chronic fatigue syndrome (CFS), recently redefined as systemic exertion intolerance disease[14], is characterized by serious fatigue that is not alleviated by rest and lasts for more than 6 months[15]. CFS is a systemic disease; in the diagnosis criteria of CDC1994[15], CFS involves multiple systems, including the nervous, digestive, and skeletal systems. CFS is accompanied by symptoms such as sore throat, tender lymphadenopathy, and impaired memory or concentration[16]. Efforts made to discover the mechanisms that cause CFS have included research on genetics, immune responses, infections, and endocrine[17]; however, despite these efforts, the cause of CFS remains unknown.

Although the etiology of CFS is still unknown, current evidence suggests that the cause of CFS involves a complex interplay between genetic, environmental, and microbial factors. CFS is a systematic disease that can occur with inflammatory symptoms[18]; for example, oral mucosal inflammation is commonly described in patients with CFS[15]. While alterations of gut microbiota have been associated with CFS[19–21], alterations to the oral microbiota in CFS patients have not been studied. The oral microbiome is worth exploring because this microbial community may contribute to CFS symptoms and because oral microbiome bacteria may be a source of noninvasive biomarkers for CFS. Therefore, to understand whether alterations occur in the oral microbiome of CFS patients, we conducted a comprehensive assessment of oral microbiome community composition and individual taxon abundance by bacterial 16S rRNA gene sequencing in CFS patients and matched controls. In addition, an analysis performed using PICRUSt[22] predicted the bacterial metagenome functional content from the 16S rRNA gene survey. This study will reveal alterations in oral microbiome composition and function in CFS patients in the Chinese Han population.

## Materials and methods

### Volunteer recruitment and sample collection

CFS patients were recruited from Southwest Hospital outpatients that fulfilled the Fukuda criteria of CFS [1] from January 2015 to April 2015. Patients with medical illnesses such as epilepsy, inflammatory bowel disease, type 1 diabetes, chronic obstructive pulmonary disease, psoriasis, rheumatoid arthritis, and lupus erythematosus were excluded. Age, sex, and body mass index were matched in healthy controls that were recruited from the Chongqing Physical Examination Center. Healthy control subjects were also excluded based on the following criteria: subjects who had similar symptoms to a CFS diagnosis, and subjects with viral infections, active bacterial, fungal, or oral disease such as gingivitis and/or periodontitis. Healthy control subjects had not taken antibiotics, probiotics, prebiotics, or synbiotics in the previous 2 months before oral saliva samples were collected.

Participant’s saliva samples were collected in a sterile 1.5 mL tube as previously described[23] and within 15 min of preparation, were stored at -80°C for further sequencing analysis. To collect unstimulated saliva and obtain sufficient numbers of bacteria, participants were asked to collect saliva in their mouth for 3 min and then drool into a tube. The study was performed according to the Declaration of Helsinki and approved by the Institutional Ethics Committee at the Third Military Medical University (Chongqing, China). Written informed consent was obtained from all participants before enrollment in this study.

### DNA extraction, bacterial 16S rRNA gene amplification, and sequencing analysis

The frozen oral saliva samples were thawed and processed using the Soil DNA Kit (Omega Bio-tek, Norcross, GA, U.S.) according to the manufacturer instructions. PCR amplification of the 16S rRNAV3-V4 hypervariable regions was performed using primers 338F (5'- ACTCCTACGGGAGGCAGCA-3') and 806R (5'- GGACTACHVGGGTWTCTAAT-3')[24]. Equimolar concentrations of purified amplicons were pooled and used for paired-end sequencing (2 × 300) on an Illumina MiSeq platform according to the standard protocols at Shanghai *Majorbio* Bio-pharm Technology. Raw sequence reads were deposited into the NCBI Sequence Read Archive (SRA) database (accession number: SRP120025, URL: https://www.ncbi.nlm.nih.gov/sra/?term=SRP120025%5BAccession%5D).

### Bioinformatics and statistical analysis

Raw data obtained from the sequencer were demultiplexed and quality-filtered using QIIME (version 1.9.1)[25]. First, the reads were truncated at any site that received an average quality score < 20 over a 50 base pair sliding window. Second, primers were matched to allow only two nucleotide mismatches, and reads containing ambiguous bases were removed. Third, sequences that overlapped for a length longer than 10 base pairs were merged according to their overlap sequence. Operational taxonomic units (OTUs) were clustered with 97% similarity cutoff using UPARSE (version 7.1 http://drive5.com/uparse/). Subsequently, chimeric and single sequences were identified and removed using UCHIME. The taxonomy of each 16S rRNA gene sequence was analyzed by RDP Classifier algorithm against the Human Oral Microbiome Database reference set (version 13) using a 70% confidence threshold.

Based on the OTUs table, diversity indices (Shannon, Simpson) and coverage were calculated using Mothur[25] and R software. Permutational MANOVA ('Adonis' function, vegan package, R)[26] of the unweighted UniFrac distance was used to test differences in overall oral microbiome composition between CFS patients and healthy controls. Violin graphs and heatmap diagrams were generated to illustrate bacterial community diversity and composition using R software (ggplot2 package). Composition analysis was used to quantify the relative abundance at the phylum and genus levels. Bacterial OTUs assigned with one sequence read were removed prior to composition analysis. Linear discriminant analysis effect size (LEfSe)[27], which identifies the differences between two or more groups by both statistical significance and biological relevance, was adopted using the Kruskal-Wallis sum-rank test to find features with significant differential abundance between groups. LEfSe analysis was performed online (http://huttenhower.sph.harvard.edu/galaxy) with an alpha value of 0.05 and a threshold on the logarithmic linear discriminant analysis (LDA) score for discriminative features of 3.0 as the critical value for all biomarkers. Two-sided p-values of 0.05 were adopted for statistical significance.

We adopted a machine-learning approach to identify CFS patients from healthy controls. For these analyses, we used taxon abundances based on OTUs at different levels. Logistic regression was used to classify CFS patients and area under the curve (AUC) was used to evaluate the performance; all these calculations were completed in the software package R. We used PICRUSt[22] to predict bacterial metagenome content from 16S rRNA gene-based microbial compositions and make functional inferences from the Kyoto Encyclopedia of Gene and Genomes (KEGG) [28] catalog. Statistical analysis of metagenomic profiles (STAMP)[29] was used to find differences in KEGG functions between the two groups. Spearman's rank correlation was used to examine associations between KEGG pathways and genera significantly associated with CFS patients (LDA > 3.0). All statistical tests were two-sided, and a p-value < 0.05 was considered statistically significant. All analyses were carried out using R software 3.3.0.

## Results

### Participants for studies of oral bacteria

Among the 91 participants (46 CFS patients and 45 healthy controls), there were no differences in age (37.43 ± 7.53 vs. 36.98 ± 7.23, p = 0.31), sex (male/female) (32/14 vs. 30/15, p = 0.94), or BMI (24.53 ± 3.23 vs 23.86 ± 3.58, p = 0.35). Many patients (38/45) identified similar infectious diagnosis; for example, in CFS patients an acute, often flu-like symptom was followed by the onset and eventual CFS diagnosis. In other patients no initiating event was recorded, and their CFS onset was gradual.

### Overview of 16S rRNA sequencing on participants: Sequences, quality analyses, and taxa identified

Sequencing the 16s rRNA gene from the oral cavity of our 91 subjects yielded 2,753,405 raw sequences, which ranged from 18,615 to 44,950 reads of 420–460 base pairs with an average length of 445 base pairs. After quality trimming and chimera checking were performed, single read sequences were removed. Because the sequencing depth per sample could affect the analysis, sequencing equaling based on the minimal reads of all subjects was performed. After these quality control checks, 15,340 high-quality reads per sample remained for further analysis. The coverage in two groups for all samples was about 99.6%, suggesting that most characters have been captured and sequencing depth for the investigation of CFS-associated oral microbiota is sufficient.

### Alterations of overall composition between CFS patients and healthy controls

**Fig 1:**
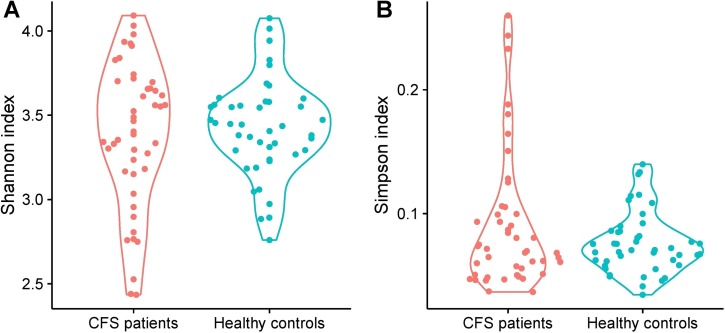
Comparison of Shannon (A) and Simpson (B) diversity indices determined for CFS patients and healthy controls.

Following sequence quality checks, bacterial richness was assessed based on the Shannon and Simpson diversity indices, which indicate the mean richness in bacterial diversity. We found similarities between CFS patients and healthy controls in their oral microbiota, but the healthy controls demonstrated slightly higher, diversity (not statistically significant) (Fig 1). To find whether the overall microbiome composition was altered in CFS patients in comparison to healthy controls, we performed a permutational MANOVA based on unweighted UniFrac phylogenetic distances. By this metric, we found a significant difference in composition between CFS patients and healthy controls (p < 0.01).

### CFS patients have differences in the relative abundances of some bacterial phyla in their oral bacterial community compared to healthy controls

**Fig 2:**
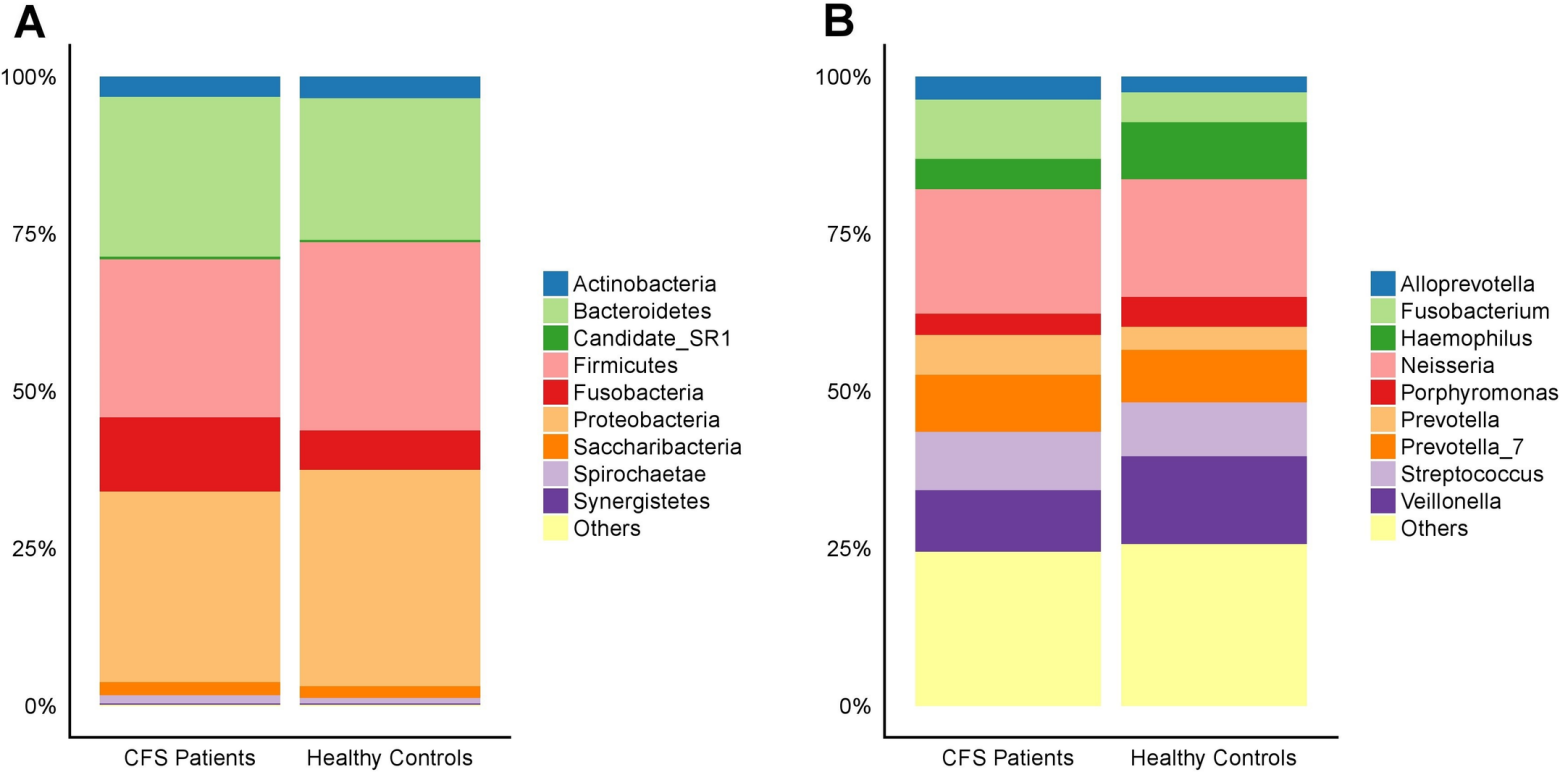
Compositions of the oral microbiota from CFS patients and healthy controls. The overall compositions of the oral microbiota from CFS patients and healthy controls are represented as bar plots at the phylum level (A) and the genus level (B).

Analyses of operational taxonomic units (OTUs) from the oral cavity of CFS patients and healthy controls revealed that their bacteria clustered within five main bacterial phyla: *Actinobacteria*, *Bacteroidetes*, *Firmicutes*, *Fusobacteria*, and *Proteobacteria* (Fig 2). However, of all the phyla with relative abundance greater than 1%, only *Fusobacteria* was altered significantly in CFS patients compared to healthy controls. Comparisons of bacterial genera between CFS patients and healthy controls uncovered results that were more complex than those observed at the phyla level. At the genus level, the five most abundant genera in CFS patients were *Neisseria* (19.77%), *Veillonella* (9.81%), *Fusobacterium* (9.39%), *Streptococcus* (9.28%), and *Prevotella_*7 (9.06%), which all together accounted for 57.31% of the oral bacterial community in CFS patients. The five most abundant genera in healthy controls were *Neisseria* (18.75%), *Veillonella* (13.97%), *Haemophilus* (9.04%), *Streptococcus* (8.52%), and *Prevotella_7* (8.35%), which accounted for 58.63% of the oral bacteria in healthy controls. Of these, *Veillonella* abundance was significantly different between CFS patients and healthy controls, with 9.81 ± 8.26% and 13.97 ± 8.91% respectively (p = 0.02) (Fig 2). In addition, many of the genera with a relative abundance greater than 1% were significantly different in CFS patients compared to healthy controls. For example, *Fusobacterium*, *Prevotella*, *Leptotrichia*, and *Campylobacter* had increased abundance in CFS patients compared to healthy controls, while *Haemophilus*, *Porphyromonas*, and *Moraxella* had decreased abundance in CFS patients compared to healthy controls.

**Fig 3:**
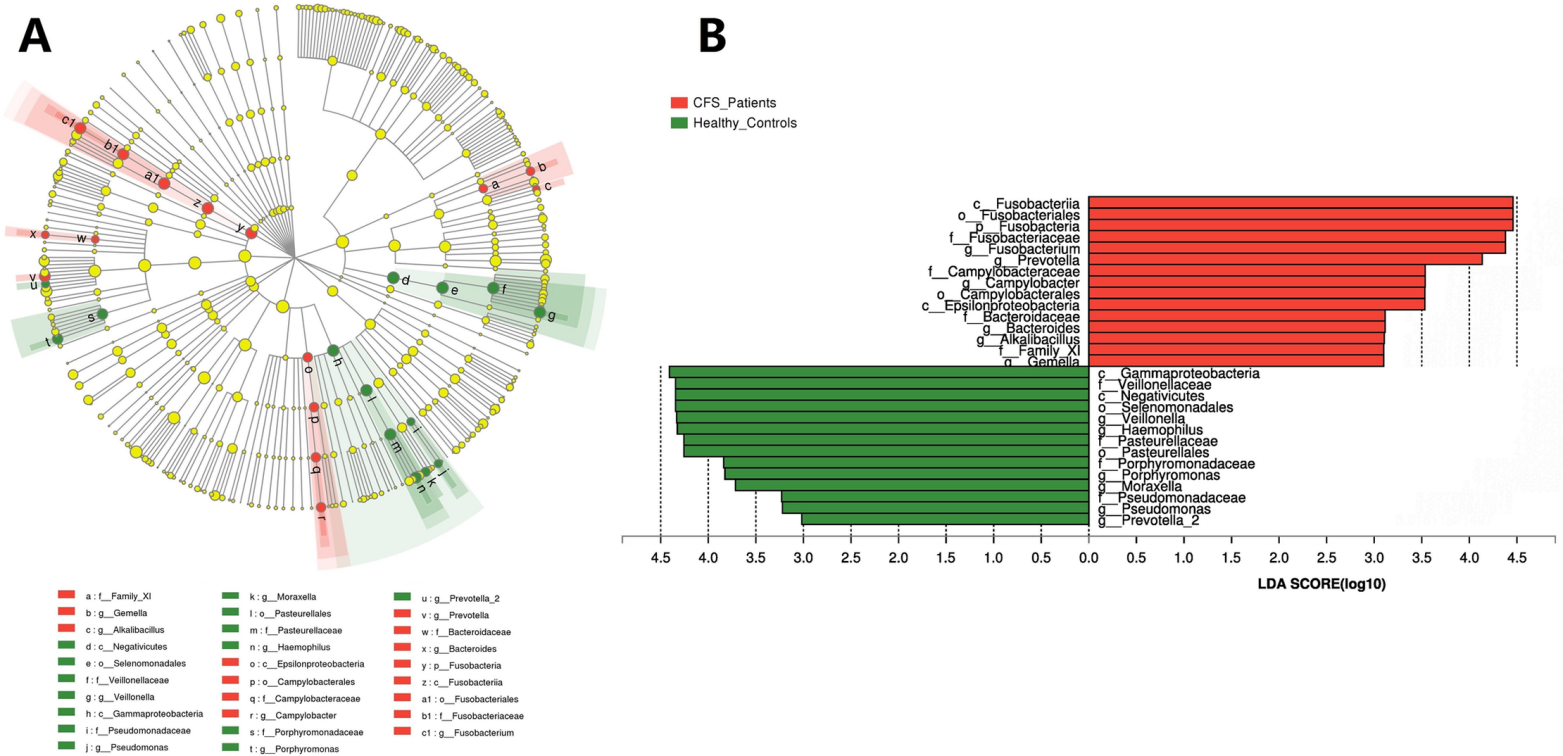
LEfSe analysis identified the most differentially abundant taxa between healthy controls and CFS patients. (A) Green indicates taxa that are enriched in healthy controls; red indicates taxa enriched in CFS patients; yellow indicates taxa that do not change between the groups. The size of each dot is proportional to its effect size. (B) Taxa enriched in the oral microbiome of CFS patients have a a positive LDA score (red), and taxa enriched in healthy controls have a negative LDA score (green). Only taxa with LDA > 3 are shown. The letter in front of the strains indicates the taxon level; p = phylum, c = class, o = order, f = family, g = genus).

We further explored these significant differences using LEfSe analysis. LEfSe analysis utilizes the Kruskal-Wallis sum-rank test to detect features in a community that have significantly different abundances, and then it employs LDA to estimate the effect size of each differentially abundant feature[27]. The output of this analysis is a cladogram that visually depicts the differences between the oral cavity microbiota of CFS patients and healthy subjects (Fig 3A), and a bar graph that represents bacteria at different levels that have an LDA score > 3.0 (Fig 3B). The LEfSe results identified the following groups as different in the oral microbiota between CFS patients and healthy controls: *Fusobacteria* in the phylum level; *Epsilonproteobacteria*, *Fusobacteriia*, *Gammaproteobacteria*, and *Negativicutes* in the class level; *Campylobacterales*, *Fusobacteriales*, *Pasteurellales*, and *Selenomonadales* in the order level; *Bacteroidaceae*, *Campylobacteraceae*, *Family_XI*, *Fusobacteriaceae*, *Pasteurellaceae*, *Porphyromonadaceae*, *Pseudomonadaceae*, and *Veillonellaceae* in the family level; *Alkalibacillus*, *Bacteroides*, *Campylobacter*, *Fusobacterium*, *Gemella*, *Haemophilus*, *Moraxella*, *Porphyromonas*, *Prevotella*, *Prevotella_2*, *Pseudomonas*, and *Veillonella* in the genus level (Fig 3).

### Machine learning and bacterial metagenome content prediction

**Fig 4:**
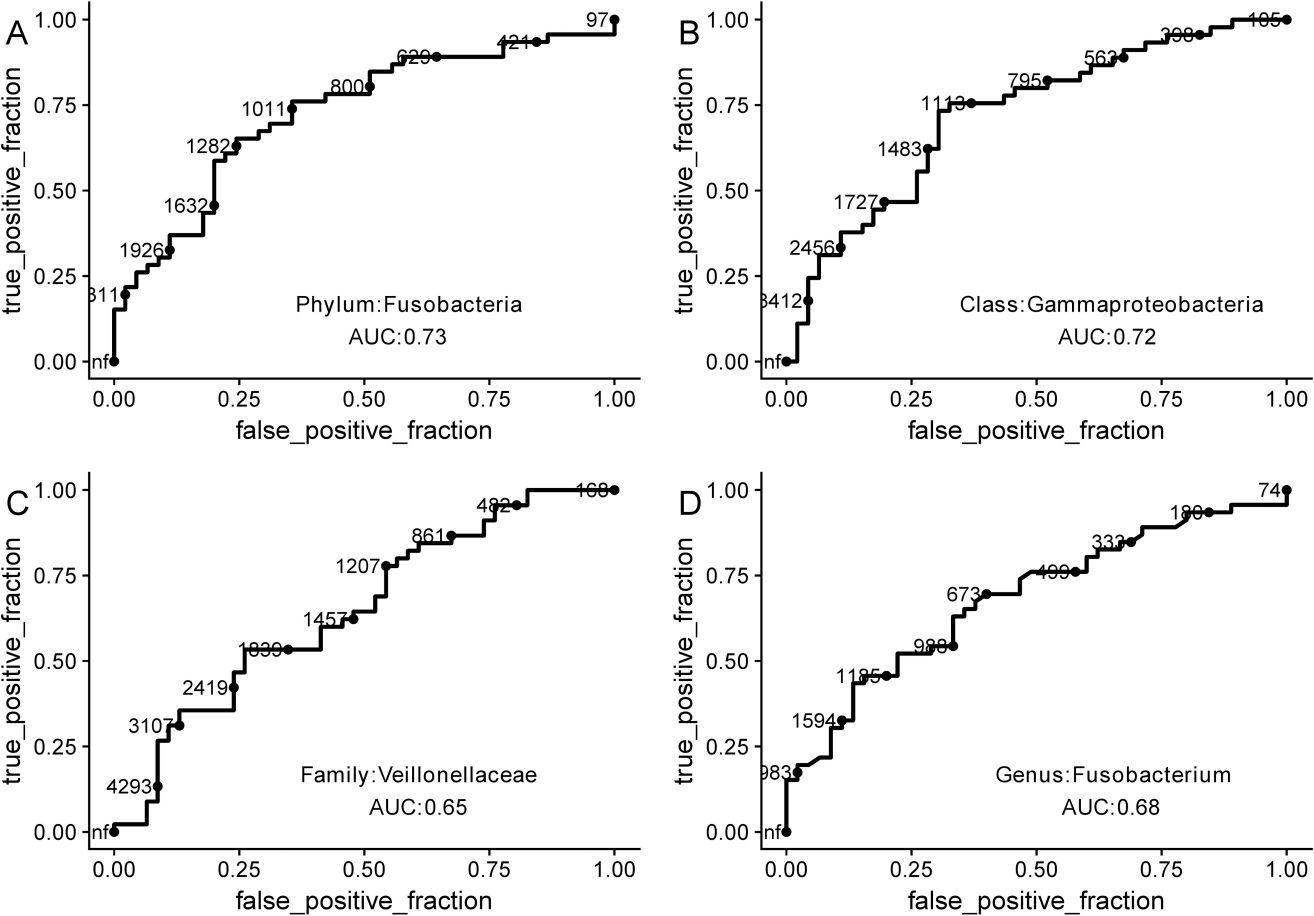
Receiver operating characteristic (ROC) curves for Fusobacteria, Gammaproteobacteria, Veillonellaceae, and Fusobacterium were used to predict CFS patients.

We wanted to further explore the diagnostic capabilities of the bacterial taxa that were significantly different between CFS patients and healthy controls. For these analyses we chose Fusobacteria, Gammaproteobacteria, Veillonellaceae, and *Fusobacterium* because they represented different taxon levels that had high LDA scores and considerable differences between groups. To assess the diagnostic ability of each taxa, we used the area under ROC (receiver operating characteristic curve) Curve (AUC). All taxa had in AUC values of around 0.7 (0.73, 0.72, 0.65, and 0.68) (Fig 4), which may indicate that a single taxon is not sufficient to diagnosis CFS. It may be that including more taxa in the analysis will increase the diagnostic capabilities of the microbiome (Fig 4).

**Fig 5:**
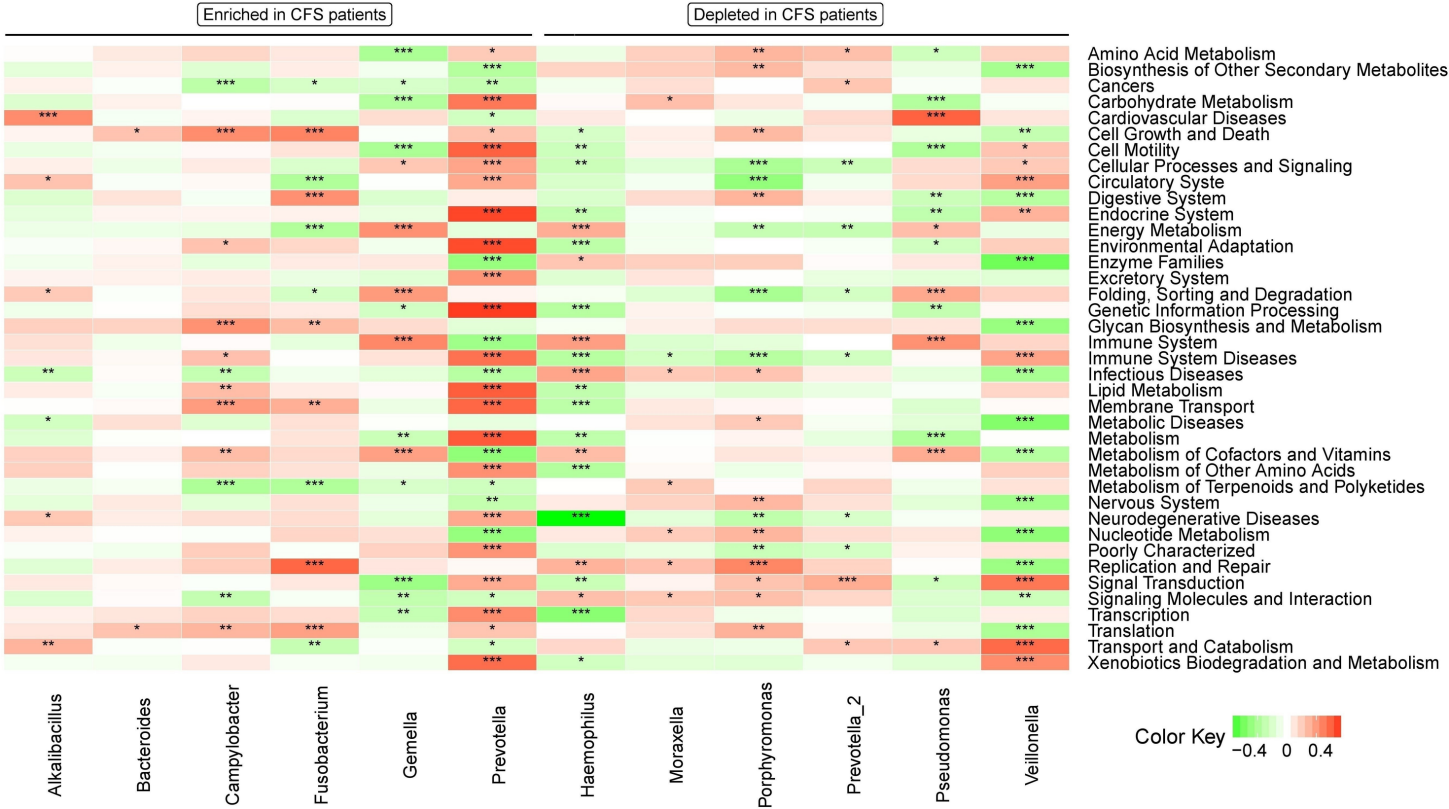
Heatmap of bacterial taxa associated with CFS that are related to several gene categories. Red indicates a positive correction, green indicates a negative correction, and white indicates no correction. *0.01 < p ≤ 0.05; **0.001< p ≤ 0.01; ***p ≤ 0.001.

To predict functions presented in the microbiome from their metagenome, we employed the PICRUSt algorithm[30]. Of the 236 KEGG pathways tested, 40 pathways differed in abundance between CFS patients and healthy controls. These 40 pathways represented different pathway levels. Specifically, no pathways in level 1, 6 pathways in level 2, and 55 pathways in level 3 were found to be significantly different between CFS patients and healthy control subjects. The level 2 pathways that distinguished CFS patients were “Biosynthesis of Other Secondary Metabolites,” “Energy Metabolism,” “Environmental Adaptation,” “Enzyme Families,” “Metabolic Diseases,” and “Metabolism of Other Amino Acids” (Fig 5). Some genera were associated with pathways in different levels; for example, *Prevotella*, which was enriched in CFS patients, was associated significantly with many level 2 pathways. *Prevotella* was positively associated with carbohydrate metabolism, cell motility, and immune system disease, and negatively associated with biosynthesis of other secondary metabolism, enzyme families, and nucleotide metabolism (Fig 5).

**Fig 6:**
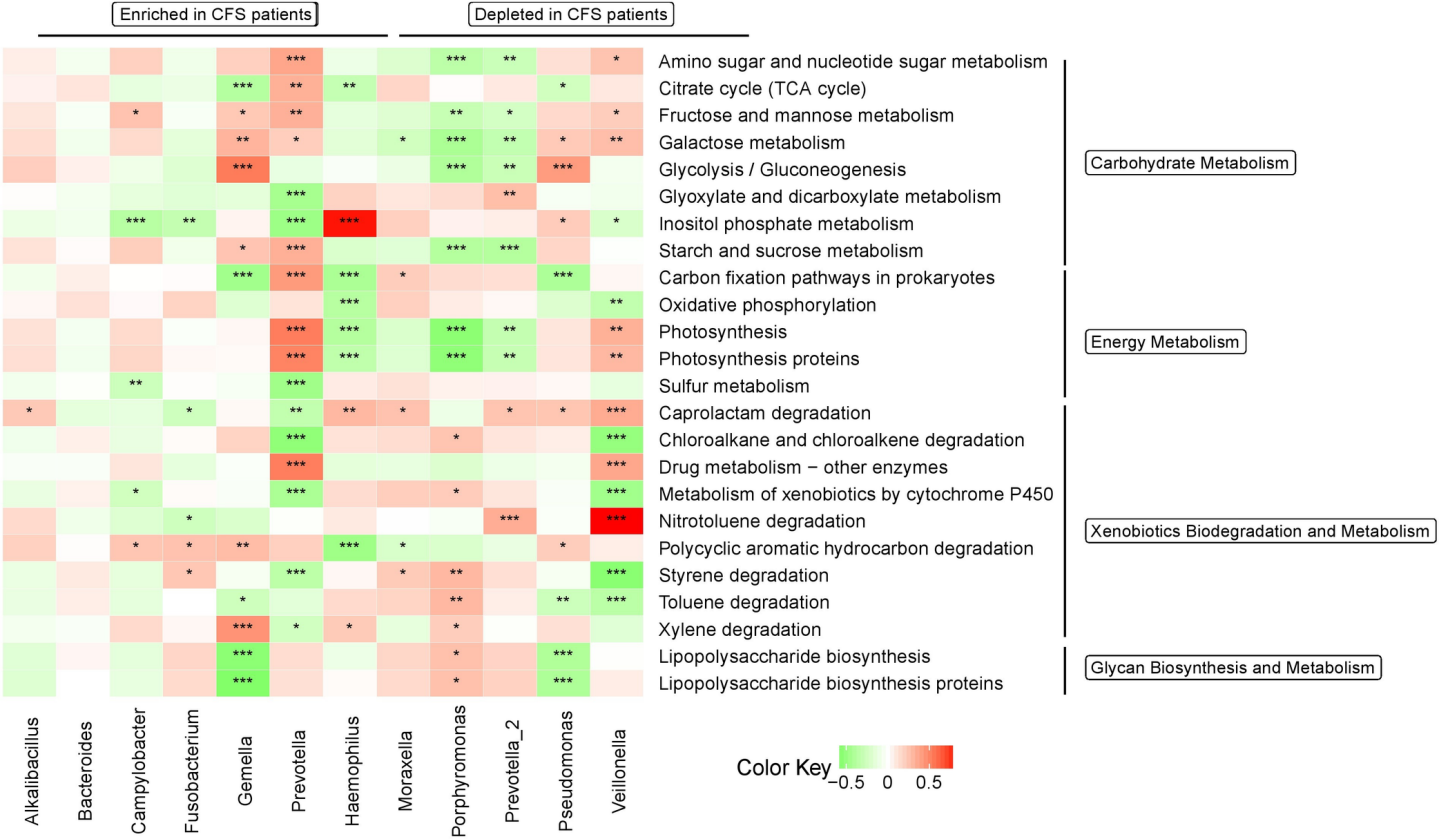
Heatmap of bacterial taxa associated with CFS that are related to several gene categories. Red indicates a positive correction, green indicates a negative correction, and white indicates no correction. Only KEGG pathways that relate to metabolism and only genera that have an LDA > 3.0 were included in the heatmap. *0.01 < p ≤ 0.05; **0.001< p ≤ 0.01; ***p ≤ 0.001.

KEGG functional pathways related to metabolism were altered in the microbiota of CFS patients. Notably, fatigue is a symptom of CFS, and metabolism is thought to play a key role in CFS pathogenesis. Therefore, we further analyzed the association between bacterial genera identified by LEfSe to have a LDA > 3.0 and KEGG pathways related to metabolism[31]. Most genera correlated with KEGG pathways that relate to metabolism (Figs 5 and 6); however, the functions in genera enriched or depleted in CFS patients were inconsistent and complex, which mirrors the complexity of CFS pathogenesis (Figs 5 and 6).

## Discussion

Due to the implication of the microbiota in human health and disease states, not only has the microbiota been a target for therapeutic interventions for a range of diseases, but therapeutic effects have been observed[32–34]. Previous studies focused on gut microbiota[19–21], which is most numerically dominant microbial community in humans. However, the oral cavity, which also teems with a large microbial community that plays an important role in human health, has recently attracted the attention of the research community.

While alterations of gut microbiota have been associated with CFS[19, 20], alterations to the oral microbiota in CFS patients had not yet been studied. Previous studies have reported that the human oral cavity microbiome is more affected by shared environment than by genetics[35], and the main phyla found in the oral microbiome are Actinobacteria, Bacteriodetes, Firmicutes, and Proteobacteria, which can vary among individuals, but corresponds to our results. Here we found, similar to studies of CFS patients’ intestinal microbiota, alterations in the oral cavity microbiota in CFS patients compared to healthy controls. Specifically, we found significant enrichment of the *Fusobacteria* phyla in CFS patients. Further, many genera were either depleted or enriched in CFS patients in comparison to healthy controls. Despite these observations, Shannon and Simpson diversity indices did not reveal differences between the two groups. We also examined associations between these altered genera and KEGG pathways and found some trends existed in the functional potential between the microbiota of CFS patients and healthy controls. Analysis of inferred metagenomes indicated that the microbiota in CFS patients may have different functions in the categories of “Biosynthesis of Other Secondary Metabolites,” “Energy Metabolism,” “Environmental Adaptation,” “Enzyme Families,” “Metabolic Diseases,” and “Metabolism of Other Amino Acids”.

Differences have been observed in the human intestinal microbiota of CFS patients[19, 20, 36]. Notably, the composition of fecal microbiota is vastly different from the composition of the oral microbiota, thus alterations we observed to the oral microbiota are substantially different from what has previously been reported for alterations to the intestinal microbiota of CFS patients. In comparison to published studies on alterations to intestinal microbiota in CFS patients, the alterations we observed in the oral cavity are less significant. For example, the intestinal microbiota of CFS patients has a significantly decreased Shannon diversity index[19] in comparison to healthy controls, which we did not observe in the oral microbiota. In addition, the specific taxa that changed in the intestinal microbiota were different from those that changed in the oral microbiota. For example, in a LEfSe analysis of CFS patients, the intestinal microbiota of CFS patients was enriched with *Oscillospira*, *Lactococcus*, and *Anaerotruncus* from the *Firmicutes* phylum, and Coprobacillus and Eggerthella from the Actinobacteria phylum. Further, 18 genera with members mainly belonging to the *Firmicutes* phylum were depleted compared to healthy controls[19]. In another study[20], the intestinal microbiota from Norwegian CFS patients had increased proportions of *Lactonifactor* and *Alistipes*, and decreased proportions of *Holdemania* and *Syntrophococcus*, while the intestinal microbiota from Belgian CFS patients showed increased *Lactonifactor* and decreased *Asaccharobacter*. Thus, CFS patients experience alterations to microbiota in both the oral cavity and intestine, but the alterations that occur in the oral cavity and intestine of CFS patients are different. However, we did find some genera whose altered proportions existed in both the oral cavity and the intestinal tract. For example, *Haemophilus* was decreased in both our oral study and Giloteaux’s study of the intestinal microbiota of CFS patients[19]. *Haemophilus*, along with *Veillonella* and *Prevotella*, have also been found altered in oral lesions[37] and in the oral microbiome of human immunodeficiency virus positive individuals[38]. While the pathogenesis of CFS remains unknown, altered microbiota, including intestinal and oral cavity, may play an important role in its etiology. Because we found many genera altered in CFS patients, the associations of these alterations and whether they all have equal significance in CFS etiology requires further exploration.

CFS is an agnogenic disease, whose symptoms involve many systems[15] and systemic inflammatory factors[18]. Thus, CFS patient oral microbiome alterations may be only one clinical index of CFS pathogenesis. It may be that oral microbiome alterations increase the severity of symptoms and thus lead to or accelerate changes in inflammatory factors. Yet the reverse may also be true, CFS-induced oral symptoms may result in oral microbiome alterations. However, regardless of the causal relationship between oral microbiome alterations and oral CFS symptoms, these two features along with alterations in inflammatory factors[18, 19] are associated with infections. Thus, infections may also contribute to CFS symptoms, although further research is required to test this hypothesis.

In addition, we found that the 16s rRNA bacterial profile of healthy controls in our study was slightly different from other published studies [38]. These discrepancies may be due to differences in subject ethnicity, and methods among others [39]. Saliva microbiome profiles are not significantly affected by the collection method or DNA extraction protocols [40], thus we matched our healthy control and CFS subjects samples in order to identify differences in their oral microbiome.

We adopted a supervised machine learning approach to identify CFS patients based on their oral microbiome, a method that has been used in several microbiome studies to distinguish patients from healthy controls[19, 41–43]. Using this approach, we classified patients with medium degree of accuracy (AUC ROC value approximately 0.7). To increase the AUC ROC value, future studies may combine inflammatory factors and intestinal microbiome with oral microbiome. The sample size is an additional confounding factor that may affect the accuracy of this analysis, along with ethnics and some methods differences[39]; thus, a large cohort of CFS and healthy controls is still needed. With these improvements, this type of approach could be further explored and serve as a complement to other non-invasive methods to distinguish patients with a variety of disease, including CFS.

We used PICRUSt to predict genomic functions present in the oral microbiome and found a difference in functions that were present in the oral microbiome of CFS patients compared to healthy controls. A correlation analysis between predicted functions and genera with LDA greater than 3.0, identified these genera to have different functions. For example, *Prevotella*, which is enriched in CFS patients, had the most significant correlations with KEGG pathways. Given how significantly *Prevotella* is altered in CFS patients, *Prevotella* may play a role in CFS pathogenesis, or acts as an indicator of oral microbiota alterations in CFS. Furthermore, of gene pathways analyzed, those related immune system and infectious diseases were significantly associated with many genera that had altered abundance in CFS patients, especially *Prevotella*, *Haemophilus*, and *Veillonella* (p ≤ 0.001). This might suggest these three genera are especially important to CFS pathogenesis.

Because fatigue is the main symptom of CFS, we were particularly interested in correlations involving pathways that related to metabolism. Analyzing these pathways in detail revealed significant differences in carbohydrate metabolism, energy metabolism, xenobiotics biodegradation and metabolism, and glycan biosynthesis and metabolism. However, we note that some changes were minor, which may be due to the complexity of CFS, how the diagnosis is based on symptoms, and how in each case the symptoms may have a different source. Despite this complexity, alterations in predicted function exist in the oral microbiome of CFS patients, and these alterations correspond to CFS symptoms—especially severe fatigue.

Our study had a few limitations, particularly related to the complex symptoms of CFS. Our study only identified oral microbiome variation between CFS patients and healthy controls but did not identify confounding factors such as disease severity, disease duration, and medical treatments received. We did not include any patients who had improved illness after treatment, or samples pre- and post-treatment, which limited conclusions we can draw from this study. Additionally, the causal relationship and mechanism of reported disease-associated microbes requires further exploration. No other studies have investigated oral microbiome alterations in CFS patients, thus further studies are required to confirm our results. While we predicted the metagenome based on our 16s rRNA gene sequencing, this analysis would have been improved with complete metagenomic data to determine the actual gene content of bacteria altered by disease. Other diseases with similar symptoms could also be included as a subgroup for future analysis to deepen our understanding of the relationship between oral microbiome alterations and CFS symptoms.

In summary, here we examined changes in the human oral microbiome in CFS patients and confirmed that the oral microbial composition of CFS patients has minor but significant differences from that of healthy controls. These genera with altered abundance are correlated with KEGG pathways, notably pathways that relate to metabolism. This study provides new insights into CFS pathogenesis, and we think that understanding the relationship between disruption of the oral microbiome and CFS symptoms may lead to improvements in treatment and diagnosis.
